# Velopharyngeal Insufficiency and Cleft

**DOI:** 10.1155/2012/864069

**Published:** 2012-11-08

**Authors:** Travis T. Tollefson, David White, James Brookes, Steven Goudy

**Affiliations:** ^1^Department of Otolaryngology-Head and Neck Surgery, University of California Davis, Davis, CA 95817, USA; ^2^Department of Otolaryngology-Head and Neck Surgery, Medical University of South Carolina, Charleston, SC 29425, USA; ^3^Department of Otolaryngology-Head and Neck Surgery, University of Calgary, Calgory, AB, Canada T2N 1N4; ^4^Department of Otolaryngology-Head and Neck Surgery, Vanderbilt University, Nashville, TN 37240, USA

Management of orofacial clefts requires a comprehensive approach to address the multifaceted aspects of possible aesthetic and functional deficits. Many children with cleft palate with or without cleft lip continue to have speech abnormalities that can affect socialization, education, and childhood development. An interdisciplinary cleft team ideally manages most orofacial clefts and can include members from the fields of facial plastic surgery, otolaryngology, speech and language pathology, pediatrics, nursing, audiology, genetics, oral surgery, orthodontics, dentistry, and social work. These teams follow a relatively established timeline and algorithm for each of the discrete steps of orofacial cleft treatment. Cleft lip repair is often performed after 3–5 months of age. Palatoplasty is delayed after 8–14 months of age. Speech and language pathology assessment and therapy are targeted as the vocabulary develops (2–5 years old).

 This special issue deals with the cleft surgeon and speech therapists role in treatment of velopharyngeal dysfunction (VPD), which is often related to velopharyngeal insufficiency (VPI). This lack of coordinated closure of the soft palate to the posterior pharyngeal wall is often a sequela of cleft palate, even after primary repair. One paper from this issue titled “*Nonlinear dynamic analysis of vowels in cleft palate children with or without hypernasality*” reports on cephalometric differences between the skull base and velopharyngeal anatomy in patients with unilateral cleft lip and palate compared to normal controls. These data contribute to the existing literature of abnormal skull base anatomy and inclination in children with velocardiofacial syndrome, which often associated with VPD.

 The next paper titled “*Noncleft velopharyngeal insufficiency: etiology and need for surgical treatment*” analyzes those children with VPD without obvious orofacial clefting, and assesses the etiologies and potential treatment options. This allows for comparison to the traditional orofacial cleft-related VPD management.

 The velopharyngeal workup is often a coordinated assessment by the cleft surgeon and speech pathologists. It can include subjective assessment, which is the subject of the next two papers. First, the next paper titled “*Nonlinear dynamic analysis of vowels in cleft palate children with or without hypernasality*” assesses the relationship between two speech assessment tools, Lyapunov exponents, and Nasalance scores in the presence of hypernasal speech. Furthermore, an additional paper titled “*Assessment of single-word production for children under three years of age: comparison of children with and without cleft plate*” presents a case-control testing of a single word assessment tool in children less than 3 years of age with cleft palate, which indentified speech differences in error patterns, consonants, articulation, and accuracy between children with and with clefts.

 Objective assessment of VPD also can include either fluoroscopic speech examination or nasopharyngoscopy to characterize the pattern of VPD and target a surgical goal. The next paper titled “*Objective assessment of hypernasality in patients with cleft lip and palate with the nasalView system: a clinical validation study*” assesses the validity and reliability of a new tool for hypernasality testing called NasalView. Speech and language therapy is instituted to address typical compensatory speech patterns seen in children with cleft palate (e.g., glottal stops). 

 If hypernasal speech is not responsive to speech and language therapy, secondary speech surgery is warranted to address hypernasality in this time period. Surgical options may include a superiorly based pharyngeal flap, dynamic sphincter pharyngoplasty, or posterior pharyngeal wall augmentation. Occasionally, a palate-lengthening procedure (Furlow double-opposing Z-plasty) is performed. These procedures can limit nasal air emissions, but potential obstructive sleep apnea from excessive nasal obstruction is a risk.

 The risk of sleep apnea after surgically treating the velopharynx may be decreased if the tonsils and adenoids are addressed preemptively. Before pharyngeal flap surgery, some surgeons perform tonsillectomy to limit the potential variability of speech and airway changes that comes with fluctuating tonsillar hypertrophy, tonsillitis, or upper respiratory infections. The use of tonsillectomy in the face of VPI in children with cleft palate is somewhat controversial and is the topic of the last paper “*Speech outcomes after tonsillectomy in patients with known velopharyngeal insufficiency*”.

 The guest editors are pleased to present a collection of papers dealing with management of the speech-related difficulties in children with orofacial clefts. In this era of the expanding role of evidenced-based medicine, the effectiveness of treatments can only be monitored and improved by assessment with valid and consistent outcome measures, which are reviewed in this issue.

*Travis T. Tollefson* *Travis T. Tollefson* *David White* *David White* *James Brookes* *James Brookes* *Steven Goudy* *Steven Goudy*

